# PLGA-Encapsulated Elvitegravir and Curcumin Modulates ART Penetration, Oxidative Stress, and Inflammation

**DOI:** 10.3390/brainsci15040328

**Published:** 2025-03-21

**Authors:** Sandip Godse, Lina Zhou, Namita Sinha, Golnoush Mirzahosseini, Santosh Kumar

**Affiliations:** 1 Department of Pharmaceutical Sciences, The University of Tennessee Health Science Center, 881 Madison Ave, Memphis, TN 38163, USA; sgodse@uthsc.edu (S.G.); lzhou13@uthsc.edu (L.Z.); nsinha2@uthsc.edu (N.S.); golnoush.mh@gmail.com (G.M.); 2 Department of Anatomy and Neurobiology, College of Medicine, The University of Tennessee Health Science Center, 875 Monroe Avenue, Memphis, TN 38163, USA

**Keywords:** HIV, HAND, BBB, elvitegravir, curcumin, PLGA, nanoparticle

## Abstract

**Background/Objectives:** HIV persists in central nervous system (CNS) reservoirs, where infected microglia and macrophages drive neuroinflammation, oxidative stress, and neuronal damage, contributing to HIV-associated neurocognitive disorder (HAND). Nanoparticle-based drug delivery systems, particularly poly(lactic-co-glycolic acid) (PLGA) nanoparticles, offer a promising strategy to improve CNS antiretroviral therapy (ART) delivery. This study aimed to evaluate the efficacy of co-administration of PLGA nanoparticles (NPs) encapsulating elvitegravir (EVG) and curcumin (CUR) in targeting CNS reservoirs, reducing neuroinflammation, and mitigating oxidative stress. **Methods:** PLGA NPs encapsulating EVG and CUR (PLGA-EVG and PLGA-CUR) were prepared via the nanoprecipitation method. The NPs were characterized for size, zeta potential, and encapsulation efficiency (EE). Their therapeutic efficacy was evaluated in vitro using U1 macrophages and in vivo in Balb/c mice. Key parameters, including cytokine levels, oxidative stress markers, and neuronal marker expression, were analyzed. **Results:** The PLGA-EVG and PLGA-CUR NPs demonstrated high EE% (~90.63 ± 4.21 for EVG and 87.59 ± 3.42 for CUR) and sizes under 140 nm, ensuring blood–brain barrier (BBB) permeability. In vitro studies showed enhanced intracellular EVG concentrations and reductions in proinflammatory cytokines (IL-1β, TNFα, and IL-18) and improved antioxidant capacity in U1 macrophages. In vivo, the co-administration of NPs improved CNS drug delivery, reduced neuroinflammation and oxidative stress, and preserved neuronal markers (L1CAM, synaptophysin, NeuN, GFAP). **Conclusions:** PLGA-based co-delivery of EVG and CUR enhances ART CNS drug delivery, mitigating neuroinflammation and reducing oxidative stress. These findings highlight the potential of nanoparticle-based ART strategies to address limitations in current regimens and pave the way for more effective HAND therapies. Future studies should focus on optimizing formulations and evaluating safety in chronic HIV settings.

## 1. Introduction

Antiretroviral therapy (ART) has been transformative in managing HIV/AIDS, significantly suppressing viral replication and reducing systemic viral loads [1]. However, HIV-associated neurocognitive disorders (HANDs) persist in up to 40% of people living with HIV/AIDS (PLWHA) on ART [2,3]. This underscores a critical limitation in current treatments: the inability of ART drugs to achieve effective concentrations in the central nervous system (CNS), primarily due to the restrictive properties of the blood–brain barrier (BBB) [4,5]. The BBB’s tight junctions, along with efflux transporters such as P-glycoprotein (P-gp) and multidrug resistance protein 1 (MRP1) and metabolic enzymes like cytochrome P450 (CYP3A4), severely limit ART penetration into the CNS, leaving viral reservoirs in the brain inadequately suppressed [6].

HIV can infiltrate the CNS early in infection via a “Trojan horse” mechanism, where infected monocytes cross the BBB, differentiate into macrophages, and establish viral reservoirs in the brain [7]. These reservoirs enable persistent low-level viral replication in CNS macrophages and microglia, even when systemic viral suppression is achieved [2]. While neurons are not directly infected due to a lack of CD4 receptors, they are highly vulnerable to indirect damage caused by viral proteins, inflammatory cytokines, and oxidative stress from HIV-infected macrophages and microglia [7]. This neurotoxic environment leads to the hallmark cognitive impairments and memory deficits associated with HAND [8].

Despite ART regimens achieving systemic viral suppression, their inability to target CNS reservoirs effectively leaves the brain vulnerable to ongoing inflammation, oxidative stress, and neurodegeneration [7,9]. Approaches to improve CNS drug delivery, such as efflux transporter inhibition, transient BBB opening, prodrug strategies, and nanoparticle (NP)-based drug delivery systems, have been investigated [9,10]. However, many of these strategies have significant limitations. For instance, blocking efflux transporters can result in off-target drug interactions, while transient BBB opening carries the risk of exposing the CNS to pathogens [10].

Nanoparticle-based delivery systems, particularly polymer-based nanoparticles (NPs) using PLGA, represent a promising alternative for improving ART distribution to the CNS [11]. PLGA NPs protect drugs from enzymatic degradation, bypass efflux transporters, and offer sustained drug release [12]. These properties make PLGA an ideal platform for delivering ART to CNS reservoirs.

Additionally, there is growing interest in combining pharmacotherapy with natural compounds, such as CUR, to enhance therapeutic outcomes [13,14,15]. CUR, derived from Curcuma longa, possesses anti-inflammatory, antioxidant, and antiviral properties, and has demonstrated neuroprotective potential in neurodegenerative disease models [16]. By reducing neuroinflammation and oxidative stress, CUR addresses key pathological features of HAND [17]. Furthermore, CUR’s ability to inhibit CYP3A4 and P-gp enhances the bioavailability and intracellular retention of co-administered drugs, making it a promising adjunct to ART [16,18].

Our previous studies demonstrated that PLGA-encapsulated elvitegravir (EVG) NPs effectively cross the BBB in vitro and in vivo, primarily via clathrin-mediated endocytosis, and reduce HIV replication in the brains of mice [19,20]. In another study, we showed that CUR enhances EVG brain delivery in Balb/c mice and in U1 macrophages improves intracellular retention, mitigates oxidative stress, and reduces inflammation [17]. Building on these findings, we hypothesized that co-delivery of EVG and CUR using PLGA NPs could additively or synergistically enhance ART efficacy in the CNS. This nanoparticle co-administration strategy aimed to increase BBB penetration, protect drugs from enzymatic degradation, and deliver therapeutic concentrations to CNS reservoirs. CUR’s antioxidant and anti-inflammatory effects are expected to complement EVG’s antiviral activity, providing a multifaceted approach to mitigate HIV neuropathogenesis and manage HAND.

In this study, we formulated PLGA NPs encapsulating both EVG and CUR (PLGA-EVG and PLGA-CUR) and characterized their physicochemical properties. We evaluated their ability to penetrate the BBB and reduce neuroinflammation and oxidative stress in in vitro and in vivo settings. The results of this study contribute to the growing body of evidence supporting nanoparticle-based ART strategies and offer new insights into potential therapies for HAND.

## 2. Materials and Methods

### 2.1. Materials

Elvitegravir (EVG, E509000) was obtained from Toronto Research Chemicals, Inc. (North York, ON, Canada) and curcumin (CUR, 78246) from Sigma-Aldrich (St. Louis, MO, USA). Poly(D, L-lactide-co-glycolide) (PLGA) (50:50 lactide-glycolide ratio, MW 31,000–50,000, ester terminated) was purchased from Birmingham Polymers (Pelham, AL, USA). Other chemicals—poly(vinyl alcohol) (PVA) (363138, 30,000–70,000), poly(L-lysine) (PLL) (MW 30,000–70,000), Pluronic F-68 (F-68) (P1300, MW 8350), and phorbol-12-myristate-13 acetate (P8139)—were purchased from Sigma-Aldrich (St. Louis, MO, USA). LC/MS-grade acetonitrile (A955) and formic acid (AC270480010), a BD PrecisionGlide 25G needle (14-826-49), and a BD 1 Ml TB syringe (14-826-88) were procured from Fisher Scientific (Hampton, NH, USA). Fetal bovine serum (FBS) was obtained from Atlanta Biologicals (Atlanta, GA, USA). Sterile phosphate-buffered saline (PBS) (10100-031) was sourced from Gibco (Dublin, Ireland). Roswell Park Memorial Institute (RPMI) 1640 media were bought from Corning Inc (Tewksbury, MA, USA). L-glutamine and penicillin–streptomycin solution were purchased from Fisher Scientific (Pittsburgh, PA, USA).

### 2.2. Animals

Male and female Balb/c mice (10weeks old) were obtained from Jackson Laboratory (Bar Harbor, MA, USA) and acclimatized for at least seven days before starting the study. Mice were housed in groups of five per cage in a sterile environment with a 12 h light/dark cycle maintained at constant temperature and humidity. Food and water were provided ad libitum throughout the experiment. Animal procedures were conducted with approval from the Institutional Animal Care and Use Committee (UTHSC-IACUC protocol 23-0464.0) and adhered to the NIH’s *Guide for the Care and Use of Laboratory Animals*. Reporting complied with ARRIVE guidelines. Thirty-six mice were divided randomly into five treatment groups—control, PLGA-control, PLGA-EVG, PLGA-CUR, and PLGA-EVG+PLGA-CUR—with three males and three females per group. The sample size was determined based on prior pharmacokinetic and biodistribution studies in mice [17,21]. Time points for sampling were selected based on the pharmacokinetics and biodistribution of EVG observed in prior studies [17]. Plasma concentrations were measured at 1, 3, 6, and 12 h due to the exponential decline of EVG, which typically becomes undetectable after 12 h. Tissue collection from the brain, liver, and lungs was performed at a terminal time point of 12 h. Mice received PLGA-EVG (25 mg/kg) and PLGA-CUR (20 mg/kg) treatments, with doses based on prior literature and experimental findings [17,22,23]. Mice were euthanized under deep isoflurane anesthesia followed by cervical dislocation. Blood was collected via cardiac puncture into EDTA-coated tubes, allowed to settle at room temperature, and centrifuged at 6000 rpm for 10 min at 4 °C to separate plasma. Tissues were homogenized in PBS (1:4 wt/vol), and plasma and brain samples were frozen at −80 °C for further analysis. For LC-MS/MS analysis, 50 µL of each plasma and tissue homogenate sample was used following established protocols with appropriate equipment and methodologies.

### 2.3. Cell Culture and Treatment

U1 cells, a chronically HIV-infected U937 cell line, were sourced from the NIH AIDS Reagent Program (Germantown, MD, USA). The U1 cell line is a well-established model for studying HIV persistence, viral latency, and macrophage-mediated infection. It provides a reproducible system for evaluating ART penetration and inflammatory responses, with data consistently validated against primary macrophages. This model effectively replicates HIV-associated oxidative stress and inflammation, making it a relevant tool for neuroHIV research [24,25,26]. The cells were maintained in RPMI 1640 medium supplemented with 10% fetal bovine serum (FBS) and 1% L-glutamine. To differentiate U1 cells into macrophages, 0.3 million cells in 0.4 mL of medium containing 100 nM phorbol 12-myristate 13-acetate (PMA) were seeded into each well of a 12-well plate. After three days of differentiation, the medium was removed and the cells washed with PBS before fresh medium was added. Differentiated cells were incubated for 3–4 h before initiating treatments. Treatment groups included a control (treated with PBS) and experimental groups exposed to PLGA-EVG (EVG: 1 µM) or PLGA-CUR (CUR: 5 µM). These concentrations, chosen to approximate physiological levels, were determined based on prior research and published studies [22,23,27]. Cells were exposed to treatments for durations specified in the protocols for each assay. Following treatment, the differentiated U1 macrophages were harvested and processed for downstream analyses according to the requirements of each experimental assay.

### 2.4. Total Antioxidant Capacity

The total antioxidant capacity (TAC) of U1 cells treated with PLGA-EVG+PLGA-CUR was evaluated using a total antioxidant capacity assay kit (Cell Biolabs, San Diego, CA, USA) following the manufacturer’s protocol. This assay measures the antioxidant potential of the samples by quantifying copper-reducing equivalents (CRE). The antioxidant capacity results are expressed in μM CRE, reflecting the overall antioxidant capability of the treated samples.

### 2.5. Cytokine Analysis

Cytokine and chemokine levels, including proinflammatory markers (IL-1β, TNF-α, IL-8, IL-6, IL-18), anti-inflammatory markers (IL-1RA, IL-10), and chemokines (MCP-1, RANTES), were quantified in the culture media of differentiated U1 macrophages and in plasma from mice. The analysis utilized Human Custom Procartaplex 9-plex and Mouse Custom Procartaplex 6-plex kits (Invitrogen, Thermo Fisher Scientific, Grand Island, NY, USA), following the manufacturer’s instructions as outlined in prior studies. Samples, standards, and magnetic beads were added to 96-well ELISA plates, mixed on a plate shaker for 1 h at room temperature, and incubated overnight at 4 °C. After incubation, the beads were washed thoroughly, and detection reagents, including the detection antibody, streptavidin–PE, and reading buffer, were sequentially added, with washing steps between each reagent. Cytokine and chemokine concentrations (pg/mL) were determined using a Magpix system, and the data were processed using xPONENT^®^ 4.2 software.

### 2.6. Western Blotting

Protein expression in mouse brain samples was assessed using Western blotting. To analyze neural marker proteins NeuN, synaptophysin, L1CAM, and GFAP, 15 μg of protein was extracted from brain homogenates of control (PBS)-, PLGA-control-, PLGA-EVG-, PLGA-CUR-, and PLGA-EVG+PLGA-CUR-treated mice. Proteins were separated on a 4% stacking and 10% resolving polyacrylamide gel for 90 min at 150 V. Following electrophoresis, proteins were transferred to a polyvinyl fluoride membrane at 0.35 A for 90 min. The membrane was blocked for 1 h with 5–10 mL of Li-Cor blocking buffer (Li-Cor Biosciences, Lincoln, NE, USA) to prevent nonspecific antibody binding. Primary antibodies were then applied overnight at 4 °C. The antibodies included NeuN rabbit polyclonal antibody (1:1000, 26975-1-AP, Proteintech Inc, (Rosemont, IL, USA)), synaptophysin mouse monoclonal antibody (1:20,000, 67864-1-Ig, Proteintech Inc, (Rosemont, IL, USA)), β-actin mouse monoclonal antibody (1:20,000, 66009-1-Ig, Proteintech Inc, (Rosemont, IL, USA)), GFAP rabbit polyclonal antibody (1:1000), and L1CAM rabbit polyclonal antibody (catalog no. 20659-1-AP, Proteintech Inc, Rosemont, IL, USA). The next day, the blots were washed three times with PBST (PBS with 0.2% Tween-20) and incubated for 1 h at room temperature in the dark with secondary antibodies: goat anti-mouse Mab (1:10,000, Li-Cor Biosciences) and goat anti-rabbit Mab (1:10,000, Li-Cor Biosciences). After washing with PBST, the membranes were scanned using a Li-Cor Scanner with Image Studio Lite version 4.0 software. Densitometric analysis was performed, and protein levels were normalized to β-actin as an internal loading control.

### 2.7. Preparation of PLGA-Based EVG/CUR NPs

PLGA NPs encapsulating EVG (PLGA-EVG), and CUR (PLGA-CUR), and blank NPs (PLGA-control) were prepared using a nanoprecipitation technique as previously described [21,28]. Briefly, PLGA (45 mg) and either EVG or CUR (4 mg) were dissolved in 4 mL of acetone to create a uniform PLGA–drug solution. This solution was added dropwise into 10 mL of 1% PVA aqueous solution while stirring at 400 rpm on a magnetic stirring plate. After 3 h of stirring, 10 mg of poly-L-lysine (PLL) was dissolved in 1 mL of water, and 50 mg of Pluronic polymer F-68 was dissolved in 4 mL of water. These solutions were added to the nanoparticle suspension and stirred for approximately 24 h at room temperature to allow complete acetone evaporation. Larger aggregates of PLGA, PLGA-EVG/CUR, PVA, and PLL were removed by centrifugation at 1000 rpm for 10 min. The resulting uniform PLGA–drug NPs were stored at 4 °C until further use.

### 2.8. Characterization of PLGA Nanoparticles

The particle size and zeta potential of the PLGA-EVG NPs were assessed using dynamic light scattering (DLS) with a Zetasizer (Nano ZS, Malvern Instruments, Malvern, UK). For particle size analysis, freshly prepared PLGA–drug nanoparticle suspensions were diluted to 1 mg/mL with distilled water and sonicated for 30 s in a Branson Ultrasonics™ 2800 water bath sonicator (Brookfield, CT, USA). Measurements were conducted in water over a 3 min interval at 25 °C. Zeta potential was measured after diluting the nanoparticle suspensions to 1 mg/mL with 1× phosphate-buffered saline (PBS).

### 2.9. Determination of Drug Loading and Encapsulation Efficiency

To measure EE, 1 mL of the PLGA NP formulation was mixed with 4 mL of acetone and allowed to stand for at least 2 h at room temperature to extract the drug. For drug loading (DL) capacity, 1 mL of the PLGA–drug formulation was freeze-dried overnight using a Labconco Freeze Dry System (−48 °C, 133 × 10⁻^3^ mBar; Labconco, Kansas City, MO, USA). The lyophilized formulation was then reconstituted in 4 mL of acetone to extract EVG or CUR. To ensure complete extraction, the acetone suspension was placed on a Corning LSE digital microplate shaker (Tewksbury, MA, USA) and gently shaken at 80 rpm for 24 h at room temperature. The supernatant was collected and diluted at a 1:300 ratio in acetonitrile for further analysis. CUR concentrations were measured using UV-vis absorbance at 450 nm (Cytation 5, BioTek, Winooski, VT, USA) as described previously [28], while the EVG concentration was determined by LC-MS/MS.

### 2.10. EVG Quantification Using LC-MS/MS

The concentration of EVG in mouse plasma, tissue samples, and cell lysates was determined using a validated LC–MS/MS method, as described previously [17,29]. The analytical system comprised a Shimadzu liquid chromatographic system (Kyoto, Japan) paired with an AB SCIEX Triple Quad 5500 tandem mass spectrometer (Framingham, MA, USA). Chromatographic separation of EVG was carried out on an Xterra^®^ MS C18 column (125 Å, 3.5 μm, 4.6 mm × 50 mm; Waters, Milford, MA, USA). The mobile phase consisted of (A) water with 0.1% formic acid and (B) acetonitrile with 0.1% formic acid, delivered at a flow rate of 1 mL/min using a gradient elution program: 50% B for 0–1.5 min and 60% B for 1.5–5.1 min. EVG and the internal standard (IS) were eluted at 3.27 and 2.72 min, respectively. Quantitative analysis was performed using multiple reaction monitoring (MRM) transitions: 447.9/343.8 for EVG and 721.3/296.1 for the IS. To extract EVG, plasma (200 μL) was mixed with 4 volumes of cold acetonitrile containing 50 ng/mL ritonavir (RTV) as an internal standard. Similarly, liver, lung, and brain samples were processed with cold methanol containing 50 ng/mL RTV. For media and cell lysates, 50 μL samples were mixed with 3 volumes of cold acetonitrile containing RTV (50 ng/mL). All mixtures were vortexed and centrifuged at 10,000 rpm for 10 min to remove proteins, and the clear supernatants were analyzed via LC–MS/MS. Calibration curves were prepared using control cell lysates, plasma, or tissue samples to account for matrix effects, ensuring accurate quantification. To determine intracellular EVG concentrations in U1 macrophages, a validated LC–MS/MS method was employed using RIPA buffer to minimize matrix effects. A calibration curve spanning 50–2000 ng/mL demonstrated excellent linearity (r^2^ = 0.999) with a weighting factor of 1/x^2^, enabling precise EVG quantification.

### 2.11. Statistical Analysis

All graphs and statistical analyses were performed using GraphPad Prism 10 (GraphPad Software; La Jolla, CA, USA). The statistical significance between two groups was determined by Student’s *t*-test. The statistical significance among treatment groups with individual variable was determined by one-way ANOVA and Tukey HSD post hoc test, which assumed that data were normally distributed with equal variance.

## 3. Results

### 3.1. Characterization of PLGA-Drug NPs

The prepared PLGA NPs were characterized for particle size, zeta potential, and polydispersity index (PDI) (Table 1). Previous studies from our lab demonstrated that PLGA NPs encapsulating EVG and DRV exhibit favorable morphological characteristics [21,29]. Similarly, Yallapu et al. encapsulated CUR into PLGA NPs using a nanoprecipitation method with PVA and PLA stabilizers [28]. Following a similar protocol, we formulated PLGA-encapsulated EVG and CUR NPs. PLGA served as the core matrix for encapsulating EVG and CUR, while layers of PVA and PLL were added to improve drug EE. Pluronic F-68 was incorporated to ensure a stable suspension suitable for therapeutic use.

Dynamic light scattering (DLS) analysis of blank PLGA NPs (PLGA-control) revealed an average particle size of 114 ± 0.79 nm. The addition of drugs slightly increased particle sizes, with EVG-loaded PLGA NPs (PLGA-EVG) measuring 125 ± 2.54 nm and CUR-loaded PLGA NPs (PLGA-CUR) measuring 138 ± 6.23 nm. Zeta potential measurements indicated low values for all formulations: −1.057 ± 0.31 mV for PLGA-control, −2.406 ± 0.84 mV for PLGA-EVG, and −1.533 ± 0.09 mV for PLGA-CUR. NPs within the size range of 100–200 nm are known to evade clearance by the reticuloendothelial system, thereby prolonging circulation times compared to free drugs [30,31]. The measured particle size and zeta potential of the PLGA–drug NPs were within the recommended range for effective drug delivery [32]. The EE and DL capacity of the NPs were also determined. EVG encapsulation showed an EE% of 90.63 ± 4.21 and DL of 2.86%, while CUR encapsulation had an EE% of 87.59 ± 3.42% and DL of 1.93. These results confirmed optimal encapsulation for the PLGA–drug nanoformulations.

### 3.2. Effect of PLGA NPs on Cytotoxicity and Antioxidant Capacity in U1 Macrophages

**Figure 1:**
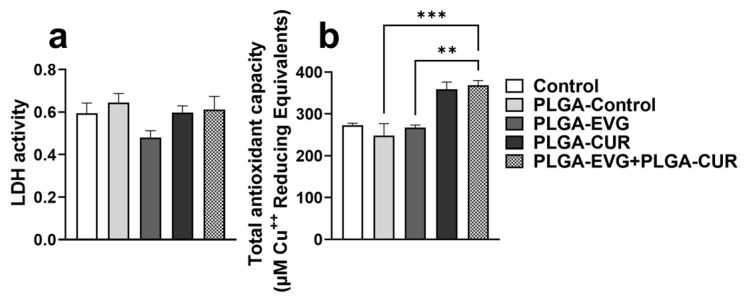
LDH activity and total antioxidant capacity. Differentiated U1 macrophages were treated with PLGA-EVG (1 µM) and PLGA-CUR (5 µM). (**a**) The lactate dehydrogenase (LDH) release in the supernatant cell media was measured at 24 h using the LDH assay kit. (**b**) The total antioxidant capacity of the cells was assessed using the total antioxidant capacity assay kit. The *Y*-axis values indicate the total amount of reduced Cu⁺ (µM), providing a quantitative measure of the cells’ antioxidant capacity. Statistical analysis was performed using one-way ANOVA followed by Tukey’s post hoc test for multiple group comparisons. Data are presented as means ± S.E.M (*n* = 4). ** *p* < 0.01, *** *p* ≤ 0.001.

An LDH cytotoxicity assay was conducted to evaluate whether PLGA NP treatments induced cell death. LDH release into the culture medium served as a marker of cell membrane damage and cytotoxicity. U1 macrophages, a monocyte-derived cell line infected with HIV, were treated with control (PBS), PLGA-control, PLGA-EVG, or PLGA-CUR for 24 h. One-way ANOVA revealed no significant differences in LDH levels across the groups, as shown in Figure 1, indicating that PLGA-control, PLGA-EVG, and PLGA-CUR treatments did not cause cytotoxicity in U1 macrophages within 24 h.

Furthermore, the total antioxidant capacity of treated cells was assessed by measuring copper-reducing equivalents. PLGA-control and PLGA-EVG treatments showed no notable effect on TAC compared to the control group. However, PLGA-CUR treatment resulted in an increase in antioxidant capacity, although the change was not statistically significant. The combination treatment of PLGA-CUR with PLGA-EVG significantly enhanced TAC compared to PLGA-EVG alone (** *p* ≤ 0.01) and the PLGA-control group (*** *p* ≤ 0.001), as shown in Figure 1.

### 3.3. Effect of PLGA NPs on Cytokines and Chemokines in U1 Macrophages

**Figure 2:**
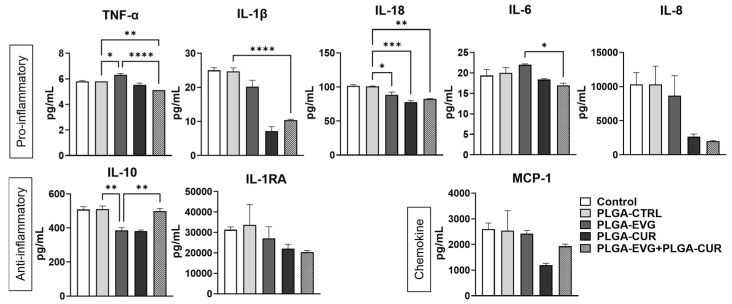
Effect of PLGA-EVG in presence of PLGA-CUR on modulation of cytokines in U1 cells. U1 cells were treated with a single dose of control, PLGA-EVG (1 µM), or PLGA-CUR (5 µM), and the levels of various cytokines and chemokines in the culture media were analyzed on day 1 using the Human Custom Procartaplex 9-plex assay. Statistical analysis was performed using one-way ANOVA followed by Tukey’s post hoc test for multiple group comparisons. Results are expressed as means ± S.E.M (*n* = 4). *, **, ***, **** *p* ≤ 0.05, *p* ≤ 0.01, *p* ≤ 0.001, *p* ≤ 0.0001 respectively.

Monocytes and macrophages are critical components of the innate immune system, playing a significant role in responding to HIV infection through the release of proinflammatory cytokines and chemokines [33]. To investigate the effect of PLGA-CUR combined with PLGA-EVG, we measured proinflammatory cytokines (IL-1β, TNFα, IL-6, IL-8, IL-18), anti-inflammatory cytokines (IL-10, IL-1RA), and the chemokine MCP-1 in the media from U1 macrophages treated for 24 h. The PLGA-control treatment showed no significant changes compared to the control group. In direct treatment, the combination of PLGA-EVG and PLGA-CUR significantly reduced proinflammatory cytokines TNF-α, IL-1β, and IL-18 compared to PLGA-control (Figure 2, ** *p* ≤ 0.01, **** *p* ≤ 0.0001). While IL-6 and IL-8 also showed a decreasing trend, the changes were not statistically significant. Similarly, MCP-1 levels were reduced with the combination treatment, though this reduction did not reach significance. It was also observed that PLGA NPs showed variable effects in the levels of anti-inflammatory cytokines IL-10 and IL-1RA, where in the case of IL-1RA, PLGA NP combination treatment did not show a significant effect, but the perceived trend showed reduction in the levels of IL-1RA with the combination treatment. In the case of IL-10, PLGA-EVG decreased the levels when compared to PLGA-control, and this decrease was resuscitated by the PLGA-EVG and PLGA-CUR combination (Figure 2, ** *p*  ≤  0.01).

These results suggest that PLGA-EVG+PLGA-CUR decreases inflammatory response in U1 macrophages by reducing proinflammatory cytokines and chemokines while increasing anti-inflammatory cytokines. Thus, this combination therapy is likely to decrease systemic inflammation in brain macrophages.

### 3.4. Effect of PLGA-CUR on Intracellular EVG Concentration in U1 Macrophages

**Figure 3:**
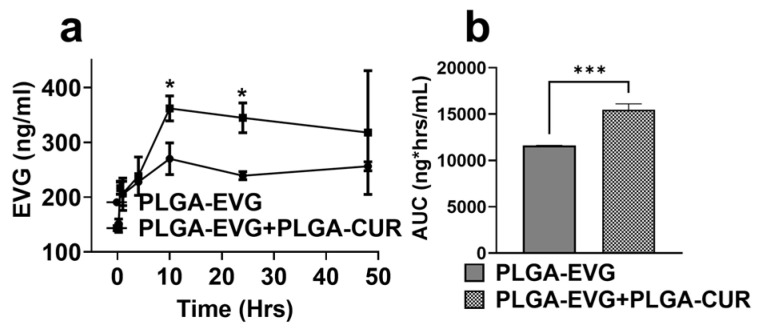
(**a**) Concentration–time profiles for the accumulation of EVG in U1 cells upon treatment with PLGA-EVG (1 µM) and PLGA-CUR (5 µM). (**b**) Area under the curve of PLGA-EVG and PLGA-EVG+PLGA-CUR. Statistical analyses were carried out using *t*-tests. Results expressed as means ± SEM (*n* = 4). *, *** *p*  ≤  0.05, *p*  ≤  0.001, respectively.

In the present study, we evaluated whether the combination of PLGA encapsulation and CUR further enhances intracellular EVG concentrations in HIV-infected U1 macrophages. In a 48 h experiment, U1 macrophages were treated with PLGA-EVG (1 µM) alone or in combination with PLGA-CUR (5 µM), and intracellular EVG concentrations were measured. The results showed significantly higher EVG levels in the PLGA-EVG+PLGA-CUR group compared to PLGA-EVG alone (* *p* ≤ 0.05), particularly at the 10 h and 24 h timepoints (Figure 3a). To evaluate overall EVG exposure, the total area under the curve (AUC_tot_) was calculated. The AUC_tot_ for PLGA-EVG+PLGA-CUR was markedly higher than for PLGA-EVG alone (*** *p* ≤ 0.001, Figure 3b). These findings highlight the ability of PLGA NPs and CUR to enhance the intracellular delivery and retention of EVG in U1 macrophages, suggesting potential improvements in EVG efficacy through this approach.

The in vitro experiments yielded the following key findings: (1) PLGA-EVG, PLGA-CUR, and their combination did not induce cytotoxicity in U1 macrophages, (2) the PLGA-EVG+PLGA-CUR combination significantly improved antioxidant capacity, (3) compared to PLGA-EVG alone, the PLGA-EVG+PLGA-CUR combination reduced inflammatory responses, and (4) PLGA-CUR enhanced the intracellular EVG concentration in U1 macrophages compared to PLGA-EVG alone. Building on these results, we conducted in vivo experiments with PLGA-EVG and PLGA-CUR formulations to ascertain these in vitro effects of PLGA encapsulation and CUR addition on in vivo experiments.

### 3.5. Effect of PLGA NP Formulations on Biodistribution of EVG via IP Route in Mice

**Figure 4:**
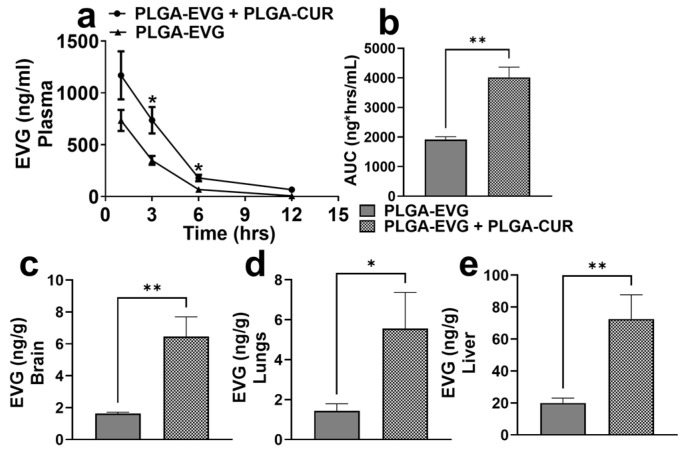
Biodistribution of EVG upon administration of PLGA-EVG in presence of PLGA-CUR via intraperitoneal route at doses of 25 mg/kg and 20 mg/kg, respectively, in Balb/c mice. EVG concentrations were analyzed in the plasma (**a**,**b**), brain (**c**), lungs (**d**), and liver (**e**). Concentrations of EVG were measured as ng/g in tissues and ng/mL in plasma. Statistical analyses were carried out using *t*-tests. Results are expressed as means ± S.E.M. (*n* = 6). *, ** *p* ≤ 0.05, *p* ≤ 0.01, respectively.

This experiment aimed to investigate the biodistribution of PLGA-EVG, both as a standalone treatment and in combination with PLGA-CUR, following IP administration. Mice were administered PLGA-EVG (25 mg/kg) alone or combined with PLGA-CUR (20 mg/kg), and the distribution of EVG was analyzed in the brain, liver, lungs, and plasma over 12 h. The plasma pharmacokinetic profile revealed that the addition of PLGA-CUR significantly increased EVG accumulation at the 3 h and 6 h time points (Figure 4a, * *p* ≤ 0.05). The plasma concentration–time profile for PLGA-EVG and PLGA-EVG+PLGA-CUR after IP administration is shown in Figure 4a. The calculated mean plasma C_max_ of PLGA-EVG+PLGA-CUR was 1168.94 ± 231.74 ng/mL, which was significantly higher than the C_max_ achieved by the PLGA-EVG (734.23 ± 101.44 ng/mL)-only administration. The AUC_tot_ of PLGA-EVG+PLGA-CUR was 4019.46 ng*h/mL, which was double that of PLGA-EVG (1933.34 ng*h/mL). The higher C_max_ and AUC_tot_ indicate that the combination NPs increased the bioavailability of EVG compared to the same dose of PLGA-EVG in mouse plasma.

Brain concentrations of EVG were measured at 12 h to assess BBB permeability. The PLGA-EVG+PLGA-CUR group showed significantly increased EVG accumulation in the brain compared to the PLGA-EVG group (Figure 4c, ** *p* ≤ 0.01). Similarly, EVG concentrations in the lungs (Figure 4d, * *p* ≤ 0.05) and liver (Figure 4e, ** *p* ≤ 0.01) were also significantly higher in the combination group. These findings highlight the potential of PLGA encapsulation combined with CUR to enhance EVG delivery across the BBB and improve its biodistribution to critical organs, supporting its use as a more effective therapeutic strategy.

### 3.6. Effect of PLGA NPs on Cytokines and Chemokines in Balb/c Mouse Plasma

**Figure 5:**
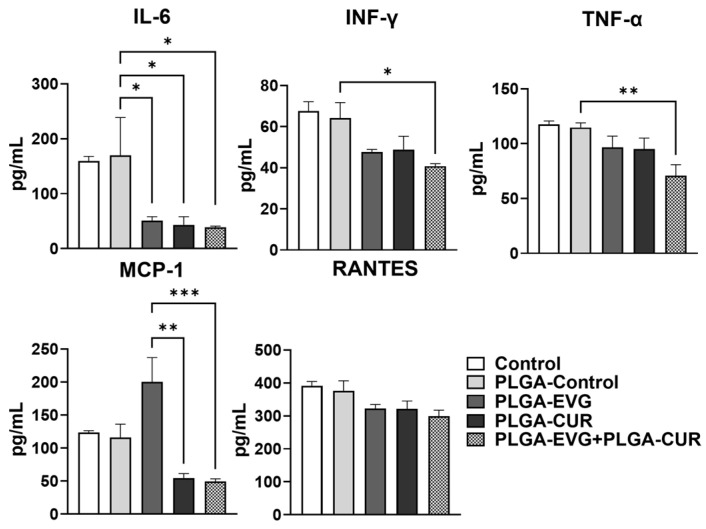
Effect of PLGA-EVG (25 mg/kg) in presence of PLGA-CUR (20 mg/kg) on modulation of cytokines in Balb/c mouse plasma. Plasma samples were prepared and analyzed by multiplex ELISA using antibodies specific for cytokines and chemokines IL-6, INF-γ, TNF-α, RANTES, and MCP-1 using Mouse Custom Procartaplex. One-way ANOVA with Tukey’s post hoc test was applied to compare between multiple groups. Results are expressed as means ± S.E.M. (*n* = 6). *, **, *** *p* ≤ 0.05, *p* ≤ 0.01, *p* ≤ 0.001, respectively.

Due to the established anti-inflammatory properties of CUR, its ability to reduce inflammation when encapsulated in PLGA NPs and co-administered with PLGA-EVG was assessed. The effect of combining PLGA-CUR (20 mg/kg) with PLGA-EVG (25 mg/kg) on the release of proinflammatory cytokines (IL-6, INF-γ, TNF-α) and chemokines (RANTES, MCP-1) was evaluated in Balb/c mouse plasma. Consistently with previous studies, blank PLGA NPs (PLGA-control) did not significantly modulate inflammatory cytokines or chemokines when compared to the control group. The results indicated that the addition of PLGA-CUR to PLGA-EVG treatment significantly reduced the level of the proinflammatory cytokines IL-6 and INF-γ (Figure 5, * *p*  ≤  0.05) when compared to the PLGA-control group. Furthermore, the results showed that the level of TNF-α suppression was more pronounced in the PLGA-EVG + PLGA-CUR treatment group than in PLGA-control (Figure 5, ** *p*  ≤  0.01). With respect to the chemokines, the levels of MCP-1 were increased in the PLGA-EVG group and significantly decreased in the PLGA-EVG+PLGA-CUR (Figure 5, *** *p*  ≤  0.001) combination group. PLGA-CUR treatment alone also showed significant modulation of anti-inflammatory cytokine IL-6 (Figure 5, * *p*  ≤  0.05) and chemokine MCP-1 (Figure 5, ** *p*  ≤  0.01).

In the case of RANTES, the overall treatment resulted in a decrease in the level, although it did not reach statistical significance in the PLGA-EVG+PLGA-CUR (Figure 5) combination group. Overall, the combination treatment with PLGA-EVG and PLGA-CUR resulted in a marked reduction in proinflammatory cytokines and chemokines compared to PLGA-control or PLGA-EVG alone. These findings indicate that the combination therapy has the potential to mitigate systemic inflammatory responses in mouse plasma effectively.

### 3.7. Effect of CUR on Neural Marker Proteins in EVG Treated Mice

**Figure 6:**
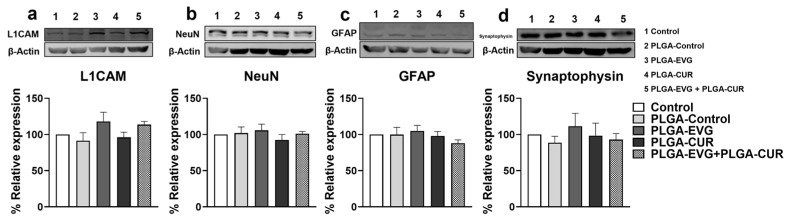
Western blot analysis was conducted to assess the expression of neural protein markers in the brains of Balb/c mice treated with PLGA-EVG (25 mg/kg) and PLGA-CUR (20 mg/kg). Proteins were extracted from brain tissue and analyzed for the expression of (**a**) L1CAM, (**b**) NeuN, (**c**) GFAP, and (**d**) synaptophysin. Data are presented as means ± S.E.M (*n* = 4). Statistical comparisons among groups were performed using one-way ANOVA followed by Tukey’s post hoc test.

HIV persistence in the CNS leads to processes such as neuronal apoptosis, disruption of neuronal support cells, and dendritic arbor loss. To evaluate the potential impact of PLGA-EVG and PLGA-CUR on neural health, we analyzed specific neuronal markers (synaptophysin, L1CAM, NeuN, and GFAP) in the brains of mice following acute treatment with PLGA-EVG and PLGA-CUR (25 mg/kg and 20 mg/kg, respectively) for 12 h. The results indicated that neither PLGA-EVG nor PLGA-CUR alone or their combination significantly altered the expression of these markers compared to the PLGA-control group (Figure 6). These findings suggest that acute exposure to PLGA-encapsulated EVG and CUR does not disrupt neural homeostasis.

## 4. Discussion

Efficient suppression of HIV in the CNS, particularly in macrophages and microglia, is crucial for the effective treatment of neuroHIV [34]. However, several challenges hinder this goal, including the limited CNS penetration of ART drugs, neuronal damage induced by HIV replication, and the potential neurotoxicity associated with ART [6]. These difficulties are compounded by the restrictive nature of the BBB, which contains efflux transporters such as P-gp and MRP1, along with metabolic enzymes like CYP3A4, further reducing ART drug concentrations in the CNS [35]. As a result, chronic inflammation and oxidative stress continue to persist in the brain, driving the progression of HAND [7].

Recent advancements in nanoparticle-based drug delivery offer promising solutions to these challenges. PLGA NPs encapsulate ART drugs, protecting them from enzymatic degradation, bypassing efflux mechanisms, and allowing sustained release [36]. PLGA NPs have shown significant potential in treating CNS diseases such as Alzheimer’s disease (AD), Parkinson’s disease (PD), ischemic stroke, and glioma [37,38,39,40]. However, their application in HAND remains limited. Our work demonstrated that PLGA-encapsulated EVG improved the suppression of HIV replication in CNS reservoirs in vitro in U1 macrophages and in vivo in Balb/c HIV encephalitis mouse brains [19,29]. Additionally, we have shown that PLGA NPs improve the permeability of DRV in U1 macrophages and wild-type mouse brains, particularly when administered intranasally [21]. Similarly, Latronico et al. reported that PLGA-encapsulated DRV improves BBB penetration and inhibits matrix metalloproteinase 9, a key factor in HAND progression [41]. These findings highlight the promise of PLGA NPs in enhancing CNS drug delivery for HAND treatment.

Curcumin (CUR), a natural compound with reported anti-inflammatory, antioxidant, and neuroprotective properties, has been investigated for its therapeutic potential in various neurological disorders [16,42]. In AD, studies suggest that CUR may inhibit amyloid plaque formation, modulate amyloid metabolism, and influence NF-κB signaling, potentially contributing to reduced neuroinflammation and cognitive improvement [43]. In PD, CUR has been associated with the enhancement of antioxidant enzyme activity (SOD, glutathione peroxidase, catalase), which could help mitigate oxidative stress and support motor function [44]. In the context of HIV-associated neurocognitive disorders (HANDs), CUR has been reported to modulate neuroinflammation, reduce ROS levels, influence autophagy, and provide some protection against gp120- and Tat-mediated neuronal damage and viral replication [45,46,47]. Additionally, its antioxidant properties, including free radical scavenging and glutathione upregulation, have been explored in oxidative stress-related conditions such as schizophrenia [47].

In this study, EVG was chosen as the therapeutic molecule for our nanoformulation due to its favorable safety profile compared to other classes of antiretroviral drugs [48], making it a suitable candidate for CNS-targeted therapy. However, EVG exhibits limited penetration across the BBB, with a cerebrospinal fluid (CSF)-to-plasma concentration ratio of approximately 0.3% in HIV-infected individuals [49]. Therefore, we developed PLGA NPs encapsulating both EVG and CUR (PLGA-EVG and PLGA-CUR), for CNS delivery. The formulation process was performed in three independent batches, and the particle size, zeta potential, PDI, and encapsulation efficiency remained consistent across all batches, indicating a high reproducibility of the PLGA nanoparticle synthesis method. The nanoprecipitation method produced NPs with high EE% (90.63 ± 4.21 for PLGA-EVG and 87.59 ± 3.42 for PLGA-CUR) and particle sizes under 140 nm with acceptable negative zeta potential, which are ideal for BBB permeability [50]. Our earlier work with EVG and DRV encapsulation in PLGA NPs demonstrated EE values of ~90% and ~84%, respectively [21,29]. Similarly, Yallapu et al. [28] reported an EE of ~90% for CUR, aligning with the current study’s findings and other reports in the literature.

Our earlier study evaluating the effect of CUR addition to the EVG treatment established that IN administration of EVG along with CUR elevates EVG concentration in the brain more effectively than IP routes of administration [17]. In this study in Balb/c mice, after dosing PLGA-EVG (25 mg/kg) along with CUR (20 mg/kg), we observed an increase in the EVG concentration in the brain in the PLGA-EVG+PLGA-CUR group compared to PLGA-EVG alone with the IP injection. In addition, EVG exposure in plasma improved about twofold in the PLGA-EVG+PLGA-CUR group compared to the PLGA-EVG group. Furthermore, combination treatment showed higher drug exposure in peripheral organs, such as liver and lungs. Although in our earlier study, CUR alone did not improve EVG penetration in the brain using the IP route [17], our results with PLGA NPs are consistent with that study, in which CUR improved EVG delivery in brain using IN administration [17]. In contrast to the current study, the IN administration of free EVG+CUR reduced the EVG levels in plasma and liver. Taken together, PLGA formulation of CUR and EVG combination enhances EVG levels in brain, plasma, and peripheral organs, indicating the role of PLGA, in addition to CUR, in enhancing EVG concentrations in the cellular targets. This conclusion is further corroborated by the findings that the PLGA formulation of EVG and CUR increased intracellular concentrations of EVG in U1 macrophages. Based on these findings, we hypothesize that PLGA-EVG+PLGA-CUR would increase stability, targeting, especially in the brain, and half-life of EVG in the plasma, which will be studied in the future.

These findings highlight the potential of co-administering PLGA-encapsulated EVG with CUR to improve CNS ART delivery. CUR may enhance EVG delivery by inhibiting the activities of CYP3A4 and P-gp, as suggested by studies showing CUR’s ability to increase the accumulation of other drugs like etoposide and tamoxifen by targeting these pathways [51,52,53]. This likely explains the increased intracellular EVG concentration in U1 macrophages observed with CUR. Additionally, CUR may facilitate EVG transport across the BBB and enhance uptake into macrophages by inhibiting P-gp, thus boosting its therapeutic efficacy [54,55]. Concurrently, PLGA encapsulation contributes by protecting EVG from enzymatic degradation, bypassing efflux mechanisms, and providing sustained drug release. Other mechanisms through which CUR increases EVG concentrations cannot be excluded, warranting further investigation. For example, considering the short half-life of CUR [56], PLGA encapsulation of CUR may protect CUR drug from enzymatic degradation, bypassing efflux mechanisms and allowing sustained release.

The limited effectiveness of current ART regimens in addressing HAND stems from their inability to adequately target the inflammatory cascades linked to neuroHIV [57,58]. Research indicates that HIV-infected individuals on ART have elevated levels of free radicals compared to untreated HIV-positive individuals or healthy controls, suggesting that both HIV infection and ART contribute to oxidative stress, exacerbating HIV pathogenesis [59,60]. CUR, known for its potent anti-inflammatory and antioxidant properties, presents a promising option as an adjuvant therapy in HIV treatment [61]. CUR also inhibits key enzymes involved in HIV replication, such as protease and integrase, and suppresses the NF-κB pathway, which is critical for HIV gene activation [62,63]. These attributes position CUR as a potential enhancer of ART efficacy.

Oxidative stress and inflammation are intricately connected, and CUR’s ability to modulate the Nrf2-Keap1 pathway underlies its dual antioxidant and anti-inflammatory effects [64]. In this study, the combination of PLGA-CUR and PLGA-EVG showed significant benefits in enhancing antioxidant capacity, perhaps leading to reduced oxidative stress, in U1 macrophages. This result suggests that while EVG alone does not contribute significantly to antioxidant activity, the presence of CUR plays a crucial role in enhancing TAC. The observed increase in antioxidant capacity with the combination treatment may be attributed to the synergistic effect of CUR in mitigating oxidative stress while ensuring sustained drug release through the nanoparticle formulation. This provides further evidence supporting the neuroprotective role of CUR in modulating oxidative stress-related pathways in the context of HIV neuropathogenesis.

Despite the effectiveness of ART, achieving full immune restoration in HIV-infected individuals remains challenging due to persistent immune activation driven by factors such as low-level HIV replication, viral proteins, microbial translocation, and co-infections [65,66]. This sustained inflammation contributes to immune dysfunction and increases the risk of inflammatory diseases like cancer, cardiovascular disorders, and neurological conditions, as highlighted by Hemalatha et al., who reported elevated inflammatory biomarkers in PLWHA on long-term ART compared to healthy individuals [67]. These findings suggest that adjuvant therapies, such as CUR, may have the potential to modulate immune activation and inflammation in ART-treated PLWHA; however, further rigorous scientific investigations are necessary to validate these effects.

Preclinical studies in in vitro and in vivo settings involving inflammatory cells and animal models have demonstrated that CUR effectively reduces proinflammatory mediators, including tumor necrosis factor α (TNF-α), interleukins (IL-6, IL-1, IL-8, IL-1β, IL-17, IL-27), RANTES, inducible nitric oxide synthase, monocyte chemotactic protein 1 (MCP-1), and granulocyte colony-stimulating factor [61]. Clinical trials further support CUR’s anti-inflammatory properties, showing reductions in markers such as C-reactive protein and TNF in people living with HIV/AIDS (PLWHA) [68]. Furthermore, in our recent study, addition of CUR to EVG treatment significantly reduced the levels of proinflammatory cytokines (TNFα, IL-1β, IL-18) and chemokines (MCP-1, RANTES) and somewhat increased the levels of anti-inflammatory cytokines (IL-10) in both U1 macrophages and wild-type mice, as discussed below [17]. The findings from the literature, including our recent study, are consistent with the findings in the current study with PLGA-EVG+PLGA-CUR. In U1 macrophages, the PLGA-EVG and PLGA-CUR combination significantly reduced proinflammatory cytokines IL-1β, TNF-α, and IL-18. Anti-inflammatory cytokine IL-10 levels were decreased by PLGA-EVG alone, but restored with the PLGA-EVG and PLGA-CUR combination. These results suggest that PLGA-EVG+PLGA-CUR improves immune response in U1 macrophages by reducing proinflammatory cytokines and enhancing anti-inflammatory activity. Similarly, in the animal study, the addition of PLGA-CUR to PLGA-EVG significantly reduced proinflammatory cytokines IL-6 and INF-γ and enhanced suppression of TNF-α. Chemokine MCP-1 levels, elevated in the PLGA-EVG group, were significantly decreased with PLGA-EVG+PLGA-CUR treatment. PLGA-CUR alone also modulated IL-6 and MCP-1. These findings indicate that the observed differences may arise from the distinct mechanisms by which EVG and CUR modulate inflammation. CUR, known for its antioxidant and neuroprotective properties, may primarily regulate inflammation by preserving homeostatic immune signaling rather than directly suppressing inflammatory cytokines. This highlights the complementary role of CUR in modulating ART-associated inflammation, which could be beneficial in mitigating HIV-related neuroinflammation while maintaining immune balance. Thus, PLGA-EVG+PLGA-CUR demonstrated improved anti-inflammatory effects, reducing systemic inflammatory responses.

Key events contributing to HAND include neuronal apoptosis, dysregulation of neuronal support cells, and loss of dendritic structure, all of which disrupt neural homeostasis [69]. Our earlier studies demonstrated that the expression of neural markers—NeuN, L1CAM, GFAP, and synaptophysin—was not significantly altered by treatment with EVG, CUR, or their combination, indicating preserved neural homeostasis [17]. NeuN, a neuronal nuclear protein involved in neurogenesis and synaptogenesis, exhibits altered localization in HAND patients [70]. GFAP, a marker for astrocytes, is upregulated in reactive astrocytes during neurological disorders, reflecting reactive astrogliosis [71]. Synaptophysin, an integral synaptic vesicle protein, regulates synaptic transmission, while L1CAM plays a critical role in neural development and regeneration [71]. Consistently with our previous results [17], the current findings revealed that PLGA-EVG, PLGA-CUR, and their combination did not significantly alter the expression of NeuN, GFAP, synaptophysin, or L1CAM compared to controls. These results suggest that neural homeostasis is not disrupted by PLGA NP treatment, supporting the safety of PLGA-EVG+PLGA-CUR in preserving neuronal function and structure.

This study has certain limitations that should be acknowledged. The U1 promonocytic cell line, derived from U937 macrophages, may not fully replicate the behavior of HIV-infected macrophages in brain reservoirs. To validate the effects of PLGA-EVG+PLGA-CUR on suppressing HIV replication, oxidative stress, and inflammation in CNS reservoirs, future studies should employ a relevant HIV mouse model. While our in vivo experiments using wild-type mice demonstrated improved brain EVG concentration with the combination PLGA nanoformulation, further investigations using an HIV-infected mouse model are crucial to determine its efficacy in suppressing HIV neuropathogenesis. Additionally, our study focused on the acute effects of PLGA-based nanoparticles; however, long-term repeated-dosing studies are required to evaluate potential accumulation, toxicity, and immune responses associated with chronic nanoparticle exposure. While PLGA nanoparticles are generally considered biodegradable and biocompatible, further research is necessary to establish their long-term safety profile in the context of chronic HIV therapy. The EcoHIV mouse model is a well-established system for studying HIV-associated neuroinflammation and cognitive deficits; however, we acknowledge that differences in brain physiology, immune responses, and ART metabolism between mice and humans could affect clinical translation. To enhance the translational relevance of our findings, future studies employing non-human primates or advanced humanized mouse models could provide deeper insights into HAND pathophysiology and nanoparticle pharmacokinetics in a human-like system.

## 5. Conclusions

To our knowledge, this is the first study demonstrating that a PLGA-encapsulated ART drug (PLGA-EVG) combined with PLGA-CUR can reduce inflammatory responses and perhaps suppress oxidative stress in U1 macrophages without causing cytotoxicity. Additionally, this study is the first to report improved CUR-mediated EVG permeability in macrophages and the brains, plasma, and other peripheral organs of wild-type mice via the IP route. These findings represent an important step toward developing PLGA-encapsulated ART with adjuvants like PLGA-CUR for treating neuroHIV and HAND. Future research will focus on applying this drug delivery system in wild-type mice for comprehensive pharmacokinetic and safety profiles and in HIV-infected mouse models to evaluate efficacy and cognitive outcomes. To enhance brain-targeted drug delivery, we propose that future studies incorporate anti-CD11b (mouse homologue of CD14) and anti-TMEM119-mAb labeling of PLGA NPs, enabling precise delivery to brain-resident macrophages and microglia. Furthermore, while PLGA is FDA-approved, we aim to explore other natural, biocompatible, and targeted NPs that are biodegradable and exhibit minimal toxicity or immune response for CNS-targeted HIV therapy.

## Figures and Tables

**Table 1:** Characterizations of PLGA NPs.

Formulation	Size (nm)	Zeta Potential (mV)	PDI	Encapsulation Efficiency (%)	Drug Loading (%w/w)
PLGA-Control	114 ± 0.79	−1.057 ± 0.31	0.241 ± 0.9	ND	ND
PLGA-EVG	125 ± 2.54	−2.406 ± 0.84	0.214 ± 1.1	90.63 ± 4.21	2.86
PLGA-CUR	138 ± 6.23	−1.533 ± 0.09	0.107 ± 2.2	87.59 ± 3.42	1.93

ND—not determined.

## Data Availability

The raw data supporting the conclusions of this article will be made available by the authors without undue reservation.
